# Redox potentials of carotenoids associated with type-II photosynthetic reaction centers

**DOI:** 10.1093/pcp/pcaf167

**Published:** 2025-12-12

**Authors:** Keisuke Saito, Hiroshi Ishikita

**Affiliations:** Department of Applied Chemistry, The University of Tokyo, 7-3-1 Hongo, Bunkyo-ku, Tokyo 113-8654, Japan; Research Center for Advanced Science and Technology, The University of Tokyo, 4-6-1 Komaba, Meguro-ku, Tokyo 153-8904, Japan; Department of Applied Chemistry, The University of Tokyo, 7-3-1 Hongo, Bunkyo-ku, Tokyo 113-8654, Japan; Research Center for Advanced Science and Technology, The University of Tokyo, 4-6-1 Komaba, Meguro-ku, Tokyo 153-8904, Japan

**Keywords:** carotenoid, photosystem II, photosynthetic reaction centers from purple bacteria, xanthophyll cycle, photoprotection

## Abstract

Carotenoids play dual roles in photosynthesis, extending light absorption and providing photoprotection through radical cation formation. Their ability to act as electron donors is defined by their redox potential for one-electron oxidation (*E*_m_). Here, we determine *E*_m_ for carotenoids whose *E*_m_ values are missing or inconsistently established in type-II reaction centers or associated antenna complexes, using a quantum chemical approach. The highest occupied molecular orbital (HOMO) energy levels correlated strongly with reported *E*_m_ values in CH_2_Cl_2_, enabling the determination of reliable *E*_m_ values. All-*trans* isomers were found to be slightly stronger electron donors than their 15-*cis* counterparts. As this *E*_m_ difference is small, the biological significance of *cis* isomerization appears to lie in pigment orientation rather than intrinsic redox properties. For xanthophyll-cycle pigments, a stepwise increase in *E*_m_ from zeaxanthin to antheraxanthin to violaxanthin, attributable to their substituents, was revealed. *E*_m_ values measured in micelles deviated markedly not only from those in CH_2_Cl_2_ but also from HOMO energy levels in water, questioning their relevance as a reference value in protein environments. These results provide a unified framework for understanding carotenoid redox chemistry in photosynthesis.

## Introduction

Photosynthesis, in which light-induced charge separation drives electron transfer reactions, is essential to sustain life on Earth. However, the same process that enables efficient solar energy conversion also produces highly reactive excited states and oxidizing species. When excitation energy exceeds the capacity of the photosynthetic reaction centers, specifically, the ability of forward electron transfer to compete, uncontrolled charge recombination and singlet oxygen formation can damage proteins, pigments, and cofactors. To preserve photosynthetic efficiency and organismal survival, photosynthetic organisms have evolved sophisticated photoprotective strategies that safely dissipate or redirect excess excitation energy.

**Figure 1:**
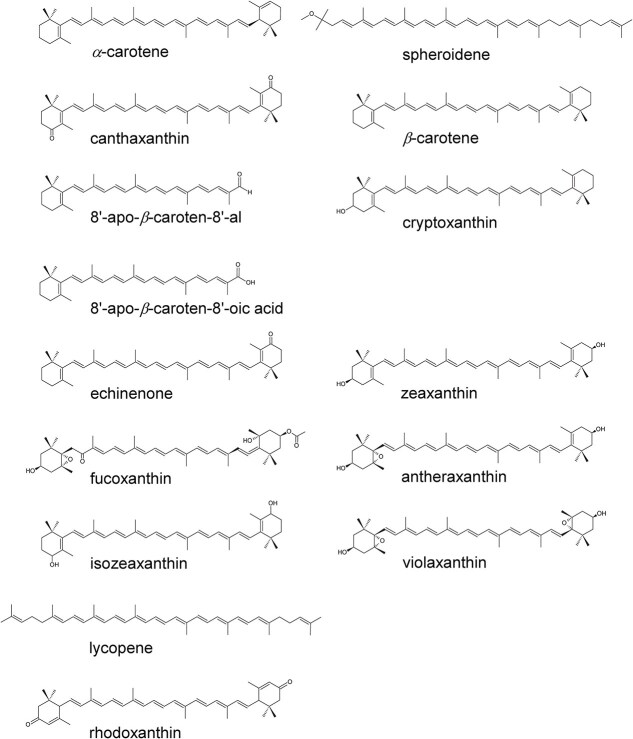
Molecular structures of carotenoids investigated. For carotenoids for which 15-*cis* isomers are also analyzed, only the all-*trans* forms are shown for clarity.

Among the central players in these protective processes are carotenoids (Fig. 1). As polyene pigments bound to photosynthetic proteins, they perform multiple functions. Carotenoids not only extend the spectral range of light absorption into the blue–green spectral region but also play a crucial role in dissipating excess chlorophyll excitation energy through energy transfer. This role is particularly critical in photosystem II (PSII), which catalyzes O_2_ evolution at the catalytic Mn_4_CaO_5_ cluster (Shen 2015), by utilizing the highly oxidizing chlorophyll cation, P680^•+^. Under conditions where forward electron transfer is inhibited, charge recombination involving P680^•+^ can lead to the formation of the chlorophyll triplet state (Rutherford and Krieger-Liszkay 2001). In particular, carotenoids efficiently quench the triplet chlorophyll state, thereby preventing the formation of singlet oxygen, a major cause of photooxidative stress. These well-established functions represent the core of the nonphotochemical quenching (NPQ) process that maintains photosynthetic efficiency under fluctuating light conditions (Horton et al. 1994, Müller et al. 2001, Polívka and Frank 2010, Hashimoto et al. 2016).

In addition to these widely accepted mechanisms, a charge-transfer quenching pathway involving carotenoid cation formation (Car^•+^) has been proposed (Galinato et al. 2007). In this model, under high-light conditions when singlet-excited chlorophylls accumulate, electron transfer from a carotenoid to a nearby chlorophyll generates a short-lived charge-separated state, Chl^•–^Car^•+^, e.g. involving a zeaxanthin cation radical (Holt et al. 2005) (Chl^•–^: chlorophyll anion radical), which rapidly recombines to dissipate excess energy as heat (Holt et al. 2005, Park et al. 2019). A similar carotenoid cation signal has been reported for all-*trans*-spheroidene in the LH2 antenna complex of purple bacteria (Polívka et al. 2002). However, the physiological significance of these mechanisms remains under debate. While the Car^•+^ signature in LHCII is experimentally reproducible (Park et al. 2018), its amplitude is small, its formation conditions are specific, and no consensus yet exists on whether it constitutes a major NPQ route (Son et al. 2021, Ruban and Saccon 2022).

In principle, carotenoid oxidation can occur readily along its π-conjugated chain (Fig. 1). Removal of an electron from the carotenoid π-conjugated chain produces a radical cation that exhibits strong absorption in the 800–1100 nm near-infrared region (Sorensen 1965, Adams 1969, Mortensen and Skibsted 1997). This spectroscopic signature serves as direct evidence that carotenoids function as electron donors under excess light conditions. A landmark study by Hanley et al. (1999) showed that illumination of Mn-depleted PSII at low temperature produces a stoichiometric Car^•+^ signal at ~990 nm, confirming that carotenoid oxidation occurs in nearly all reaction centers and can act as an electron donor to P680^•+^. Upon warming, Car^•+^ was replaced by a chlorophyll cation (Chl^•+^), indicating a sequential electron-transfer pathway (Chl → Car → P680) (Hanley et al. 1999). The chlorophyll cation responsible for oxidation of the carotenoid (Car_D2_, Faller et al. 2001) was suggested to be the accessory chlorophyll located on the D2 protein, Chl_D2_ (Faller et al. 2005). Although the redox potential values for one-electron oxidation (*E*_m_) of carotenoids, including Car_D2_, have not been reported for high-resolution PSII structures (e.g. 1.9 Å structure, Umena et al. 2011), the highest occupied molecular orbital (HOMO) of Chl_D2_ is most stable among the four chlorophylls in the D1/D2 electron transfer branches. Consequently, the *E*_m_ of Chl_D2_ is expected to be the highest (Tamura et al. 2020), higher than at least ~1000 mV (Saito et al. 2011). Since the *E*_m_ value of *β*-carotene (540 mV in CH_2_Cl_2_  *versus* the saturated calomel electrode, SCE; Liu et al. 2000) is comparable to that of chlorophyll *a* (620 mV in CH_2_Cl_2_  *versus* SCE, Wasielewski et al. 1981), the carotenoid can mediate electron (hole) transfer between chlorophyll molecules in the protein environment, where the local electrostatic field tunes the *E*_m_ value of each cofactor. Thus, it seems plausible that Car_D2_ can reduce P680^•+^ and thereby prevent the accumulation of harmful strong oxidants and reactive oxygen species (Hanley et al. 1999, Faller et al. 2001, Tracewell et al. 2001, Telfer 2002, Faller et al. 2005).

It should be noted that in the 1.9 Å structure of PSII, one carotenoid originally modeled as *β*-carotene (PDB: 3ARC) was later remodeled as cryptoxanthin (“RRX101” in PDB: 3WU2) (Umena et al. 2011), whose hydroxyl group likely interacts with the N-terminus of PsbX on the peripheral surface of the CP47 side of the PSII complex, far from the D1/D2 proteins. In contrast to all-*trans*-*β*-carotene in PSII, only a single 15-*cis*-spheroidene exists in reaction centers from purple bacteria (PbRC). Recent theoretical work suggested that 15-*cis*-spheroidene is energetically capable of forming a cationic state in PbRC (Noji et al. 2025), although this mechanism has not yet been experimentally verified.

Notably, one of the carotenoids in the early 3.7 Å PSII structure reported by Kamiya and Shen (Kamiya and Shen 2003) was modeled as 15-*cis*-*β*-carotene, the interpretation of which was likely influenced by the earlier report of Bialek-Bylk et al. (1995) that 15-*cis*-*β*-carotene had been spectroscopically identified in PSII. In that study, because *cis*-spheroidene located near the B-branch accessory bacteriochlorophyll of PbRC had been implicated in photoprotection (Tandori et al. 2001), Bialek-Bylk et al. (1995) extended this concept to PSII and speculated that 15-*cis*-*β*-carotene might play an analogous photoprotective role. However, all subsequently published PSII structures have demonstrated that 15-*cis*-*β*-carotene is not present (e.g. Ferreira et al. 2004, Loll et al. 2005, Umena et al. 2011). Irrespective of these interpretations concerning *cis*-configured carotenoids, it still remains unclear whether the *cis* forms of *β*-carotene and spheroidene are significantly more effective electron donors than their *trans* counterparts, owing to the absence of reported *E*_m_ values.

The capacity of carotenoids to form cation radicals can be assessed by their *E*_m_ values. Because hydrophobic carotenoids cannot be dissolved in water, their *E*_m_ values were extensively measured in nonpolar solvents such as CH_2_Cl_2_, mostly before the early 2000s (e.g. Park 1978, Land et al. 1987, Jeevarajan et al. 1994, Liu et al. 2000, Niedzwiedzki et al. 2005). Nevertheless, to the best of our knowledge, *E*_m_ values for several key carotenoids involved in type-II reaction centers and associated antenna complexes, sometimes in specific configurations, remain unknown (e.g. spheroidene, which exists as the 15-*cis* form in PbRC and the all-*trans* form in LH2). In addition, for some carotenoids, *E*_m_ values measured in micelles are reported, but whether these can be regarded as equivalent to “*E*_m_ values in water” remains unclear. Here, we determine *E*_m_(Car/Car^•+^) for these carotenoids lacking experimentally measured values, using a quantum chemical approach previously validated for redox cofactors (Kishi et al. 2017).

## Results and Discussion

### Correlation between calculated energies and experimentally measured *E*_m_ values

**Figure 2:**
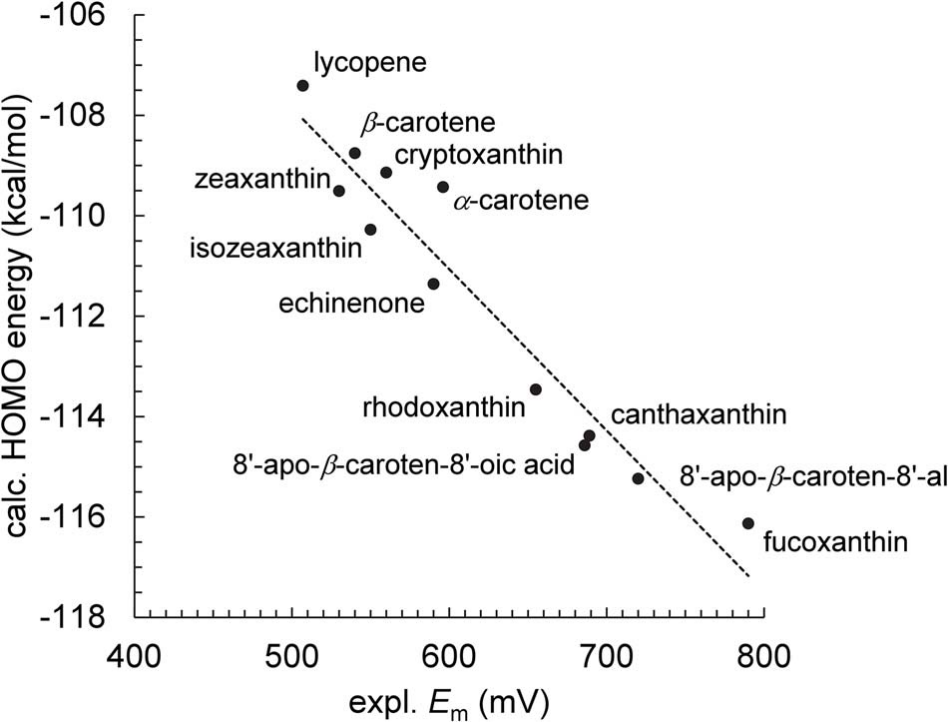
Correlation between experimentally measured *E*_m_(car/car^•+^) in CH_2_Cl_2_ (expl. *E*_m_) *versus* SCE and calculated HOMO energy levels (calc. HOMO, coefficient of determination *R*^2^ = 0.93). The solid line was drawn according to Equation (5).

The calculated *E*_HOMO_ (Supplementary Table S1), which is associated with one-electron oxidation of carotenoids (Car → Car^•+^ + e^−^), is highly correlated with the experimentally measured *E*_m_ values in CH_2_Cl_2_  *versus* SCE, *E*_m_(Car/Car^•+^), for 12 carotenoid species (Liu et al. 2000) (Fig. 2). The correlation is best described by the following linear relationship (coefficient of determination *R*^2^ = 0.93, Fig. 2):


(1) \begin{eqnarray*} && {E}_{\mathrm{m}}\left(\mathrm{Car}/{\mathrm{Car}}^{\bullet +}\right)\ \mathrm{in}\ {\mathrm{CH}}_2{\mathrm{Cl}}_2\ versus\ \mathrm{SCE}\ \left[\mathrm{mV}\right]\nonumber\\&& \qquad =\ \hbox{--} 31.2\ \left({E}_{\mathrm{HOMO}}+91.79\ \left[\mathrm{kcal}/\mathrm{mol}\right]\right). \end{eqnarray*}

Using the calculated HOMO energy levels in combination with Equation (1), it is possible to calculate *E*_m_(Car/Car^•+^) values for carotenoid molecules whose experimental values have not been reported. Note that the overall root mean square deviation between the experimentally measured and the calculated *E*_m_(Car/Car^•+^) values for the 12 carotenoid molecules is 24 mV (Table 1), comparable to the deviation reported for quinone molecules (21 mV) (Kishi et al. 2017).

**Table 1:** Calculated *E*_m_ values for carotenoids in CH_2_Cl_2_  *versus* SCE (mV). Experimentally measured values reported by Liu et al. (2000) and Niedzwiedzki et al. (2005) are included for comparison. *E*_m_ values for carotenoids in water *versus* NHE can be estimated by adding +246 mV to the *E*_m_ values in CH_2_Cl_2_  *versus* SCE (see text)

**Carotenoid**	**CH**_**2**_ **Cl**_**2**_ ***versus* SCE**		
	Liu et al. (2000)	Niedzwiedzki et al. (2005)	**Calc.**
*α*-carotene	596		550
Canthaxanthin	689		704
8′-apo-*β*-caroten-8′-al	720		730
8′-apo-*β*-caroten-8′-oic acid	686		710
Echinenone	590		610
Fucoxanthin	790		758
Isozeaxanthin	550		576
Lycopene	507		487
Rhodoxanthin	655		675
[Type-II reaction centers]			
Spheroidene			528
15-*cis*-spheroidene			555
*β*-carotene	540	567	528
15-*cis*-*β*-carotene			557
Cryptoxanthin	560		541
[Xanthophyll cycle]			
Zeaxanthin	530	571	552
Antheraxanthin			571
Violaxanthin		681	615

Importantly, in aqueous solution, the potential of SCE relative to the normal hydrogen electrode (NHE) is well established; *E*_m_(Car/Car^•+^) in “water” *versus* SCE could be converted to *E*_m_(Car/Car^•+^) in “water” *versus* NHE simply by adding ~ 240 mV. However, all experimentally measured *E*_m_(Car/Car^•+^) values have been reported in CH_2_Cl_2_  *versus* SCE (Liu et al. 2000), not in water *versus* SCE. In nonaqueous solvents, the potential difference between SCE and NHE cannot be regarded as constant because it includes an undefined liquid-junction potential and solvent-specific effects. For quinones, an apparent offset of ~480 mV between [*E*_m_ in dimethylformamide (DMF) *versus* SCE (Prince et al. 1983)] and [*E*_m_ in water *versus* NHE (Swallow 1982)], which exceeds the ~ 240 mV difference in water, appears to arise not only from the difference in dielectric constants (37 for DMF and 80 for water) but also from liquid-junction potentials, which vanish only when both scales are re-referenced to the ferrocene redox couple (Fc/Fc^+^). Even then, an intrinsic solvent-dependent shift of ~600 mV remains (Kishi et al. 2017). These issues may not necessarily hold true for all systems, including carotenoids and chlorophylls. For instance, the experimentally measured *E*_m_ value of 860 mV for chlorophyll *a* in CH_2_Cl_2_  *versus* NHE was obtained by simply adding ~240 mV to the original *E*_m_ value measured in CH_2_Cl_2_  *versus* SCE (Wasielewski et al. 1981), as is commonly done in studies on chlorophyll electrochemistry (Watanabe and Kobayashi 1991). Given these uncertainties, calculated *E*_m_ values in CH_2_Cl_2_  *versus* SCE are primarily presented in the present study, as is commonly done in studies on carotenoid electrochemistry (Liu et al. 2000, Niedzwiedzki et al. 2005).

That said, *E*_m_ values in water *versus* NHE would be useful as *E*_m_ values more relevant to protein environments. The *E*_m_ values for protonated tyrosine (Tyr-OH/Tyr-OH^•+^) and deprotonated tyrosine (Tyr-O^−^/Tyr-O^•^) are 1380 mV and 680 mV in water *versus* NHE (Tommos and Babcock 2000), and correspond to the HOMO energy levels of Tyr-OH and Tyr-O^−^, respectively. Assuming that the correlation observed for tyrosine between *E*_m_ in water *versus* NHE and the HOMO energy in water also holds true for *β*-carotene in water (Supplementary Table S1), its *E*_m_ value is estimated to be 774 mV in water *versus* NHE, 246 mV higher than the value in CH_2_Cl_2_  *versus* SCE (Table 1). Based on this estimation, tentative *E*_m_ values in water *versus* NHE for carotenoids can be obtained by adding +246 mV to their *E*_m_ values in CH_2_Cl_2_  *versus* SCE, which is substantially consistent with the typical ~ 240 mV conversion from SCE to NHE in water.

### Carotenoids associated with type-II reaction centers

**Figure 3:**
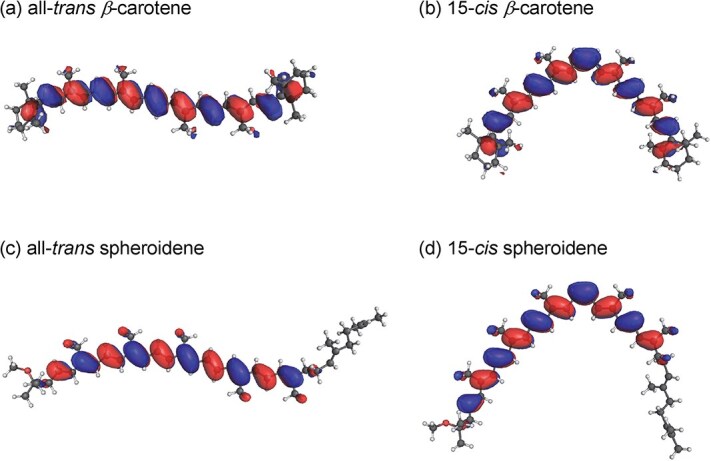
HOMO distribution in *trans*- and *cis*-carotenoids. (a) All-*trans β*-carotene, (b) 15-*cis β*-carotene, (c) All-*trans* spheroidene, and (d) 15-*cis* spheroidene.

**Figure 4:**
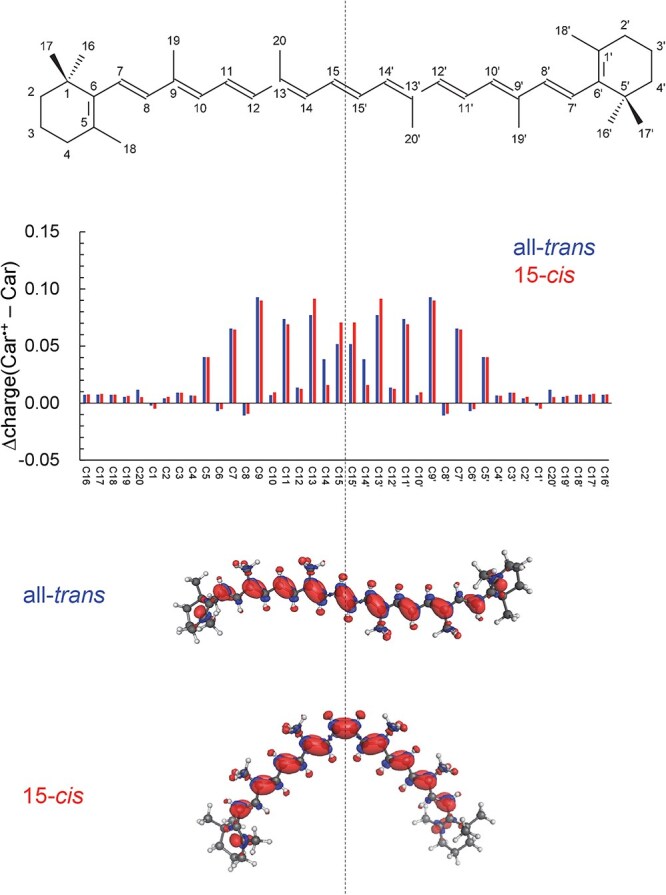
Redistribution of atomic charge and electronic density upon one-electron oxidation in *β*-carotene. (top) Molecular structures of *β*-carotene with atom numbering. (middle) Changes in atomic partial charges upon one-electron oxidation [Δcharge(Car^•+^ − Car)] for the all-*trans* (blue) and 15-*cis* (red) isomers. (bottom) Electronic density difference (Car^•+^ − Car) for the all-*trans* and 15-*cis* isomers. Red and blue surfaces represent regions of increased and decreased electron density, respectively. The vertical dotted line indicates the center of conjugation, which in this case coincides with the *cis* C15 = C15’ moiety.

The present study shows that the *E*_m_ value of all *trans*-*β*-carotene is ~30 mV lower than that of 15-*cis*-*β*-carotene (Table 1). This difference arises because the π-electron system of all-*trans*-*β*-carotene is more effectively delocalized than that of the 15-*cis* isomer, owing to the geometric distortion at the 15-*cis* double bond (Fig. 3a and b). In particular, in the *cis* form, more positive charge localizes on C15/C15’ (the central *cis* C=C) and on C13/C13’, whereas in the *trans* form more positive charge appears at C14/C14’. In the *cis* form, the closer face-to-face geometry around C15 = C15’ limits delocalization onto C14/C14’, resulting in a concentration of positive charge at C15 = C15’ (Fig. 4). Consequently, the *trans* form, in which positive charge can be more delocalized over C14/C14’, is oxidized more easily, leading to a lower *E*_m_ value (Table 1). Thus, all-*trans*-*β*-carotene is an energetically slightly stronger electron donor than the 15-*cis* isomer, contrary to their expectations (Bialek-Bylk et al. 1995), although the difference is only ~30 mV.

The same trend holds for spheroidene, where the *E*_m_ value of all-*trans*-spheroidene is ~30 mV lower than that of 15-*cis*-spheroidene (Table 1, Fig. 3c and d, and Supplementary Fig. S1). In contrast to 15-*cis*-*β*-carotene, in which the *cis* C15 = C15’ bond is consistent with the center of the conjugated system, 15-*cis* spheroidene exhibits a shifted conjugation center located between C12 and C13 (Supplementary Fig. S1). Nevertheless, the characteristic charge distribution observed for *β*-carotene is also seen for spheroidene, in the *cis* form, more positive charge localizes on the central C15 = C15’ bond and on C13/C13’, whereas in the *trans* form, positive charge preferentially accumulates at C14/C14’ (Supplementary Fig. S1). This result, in turn, suggests that the presence of a *cis* C=C bond itself, rather than the position of the conjugation center, is the primary factor responsible for the localization of positive charge on the central C15 = C15’ bond in 15-*cis*-*β*-carotene.

Because the *cis*-*trans* difference in *E*_m_ is relatively small, the physiological significance of the *cis* form is likely related more to pigment orientation than to *E*_m_ itself. In the all-*trans* form, the polyene chain tends to align parallel to the lipid acyl chains, favoring an orientation perpendicular to the membrane plane. In contrast, the 15-*cis* form, as seen for spheroidene in PbRC, promotes an orientation more parallel to the membrane plane, thereby enabling closer van der Waals contacts with the chlorin plane of the B-branch accessory bacteriochlorophyll, B_B_. In this case, furthermore, not only because of the orientation, but also because more positive charge localizes on the central *cis* C15 = C15’ bond moiety upon oxidation (Supplementary Fig. S1), the *cis* form appears to be more advantageous as an efficient electron donor to the chlorin ring of B_B_^•+^ if it forms.

A related observation was reported in resonance Raman studies of triplet-excited spheroidene in PbRC, where large changes in bond order were found in the C11 = C12–C13 = C14 portion of the polyene chain (Koyama et al. 2006). Although triplet excitation [a HOMO to lowest unoccupied molecular orbital (LUMO) transition] and one-electron oxidation (removal of an electron from the HOMO) represent different electronic processes, both analyses identify the region near C13 as particularly electronically responsive.

### Xanthophyll-cycle carotenoids

The xanthophyll cycle represents a central photoprotective mechanism in plants, in which violaxanthin is enzymatically de-epoxidized under high-light conditions to antheraxanthin and further to zeaxanthin. This de-epoxidation extends the effective conjugation length of the carotenoid, lowering its S_1_ excitation energy and thereby dissipating excess excitation energy through energy transfer from nearby chlorophylls.

To the best of our knowledge, the *E*_m_ value of antheraxanthin has not been reported. For zeaxanthin, two groups have reported divergent values: 530 mV by Liu et al. (2000) and 571 mV by Niedzwiedzki et al. (2005), differing by ~40 mV (Table 1). Moreover, the relative ordering with *β*-carotene is inconsistent: Liu et al. (2000) reported zeaxanthin to be 10 mV lower than *β*-carotene, whereas Niedzwiedzki et al. (2005) reported it to be 4 mV higher, leaving the ranking unresolved (Table 1).

**Figure 5:**
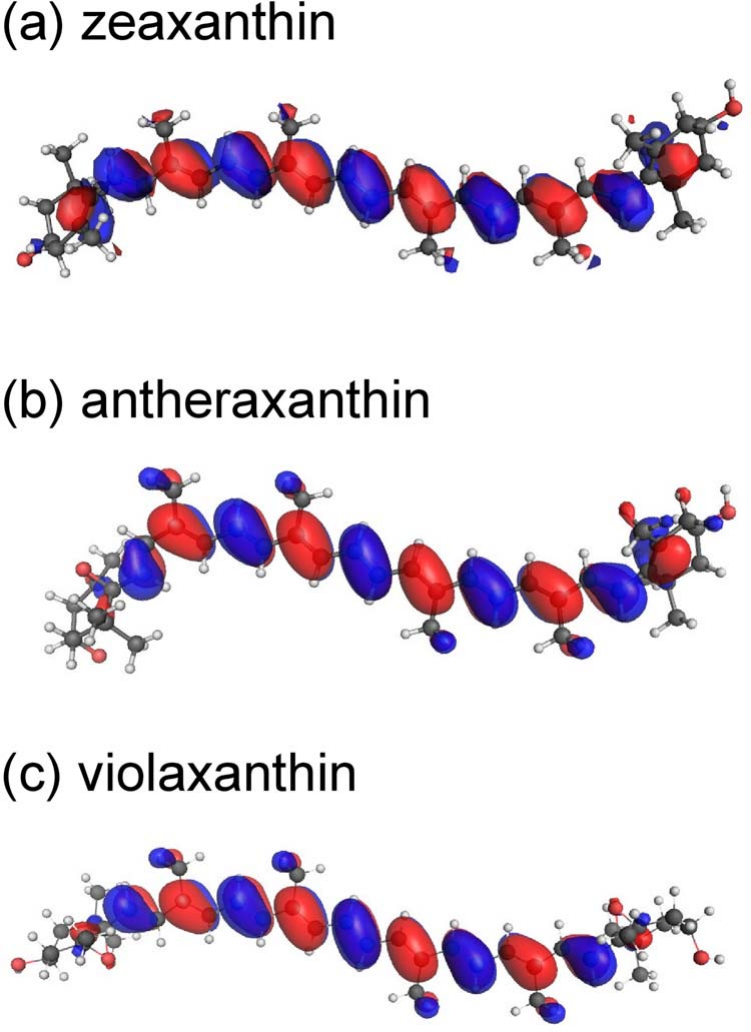
HOMO distribution in xanthophyll-cycle carotenoids. (a) Zeaxanthin, (b) antheraxanthin, and (c) violaxanthin.

The present study shows that the *E*_m_ of zeaxanthin is ~20 mV higher than that of *β*-carotene (Table 1). This reflects the presence of hydroxyl substituents, which slightly stabilize the HOMO through electron-withdrawing effects, making oxidation less favorable (Fig. 5a). The *E*_m_ of antheraxanthin is ~20 mV higher than that of zeaxanthin (Table 1), consistent with the additional epoxy group, which disrupts conjugation between the terminal rings and the polyene chain and thereby shortens the effective conjugation length. Violaxanthin, with two epoxy groups, shows a further ~40 mV increase relative to antheraxanthin (Table 1) for the same reason.

Thus, the electron-donor ability of the xanthophyll-cycle carotenoids decreases in a stepwise manner, from zeaxanthin to antheraxanthin to violaxanthin (Fig. 5), although this trend does not by itself indicate that cation radical formation (Galinato et al. 2007) is a physiologically relevant photoprotective mechanism in the xanthophyll cycle.

### *E*_m_ values in aqueous micellar environments

**Figure 6:**
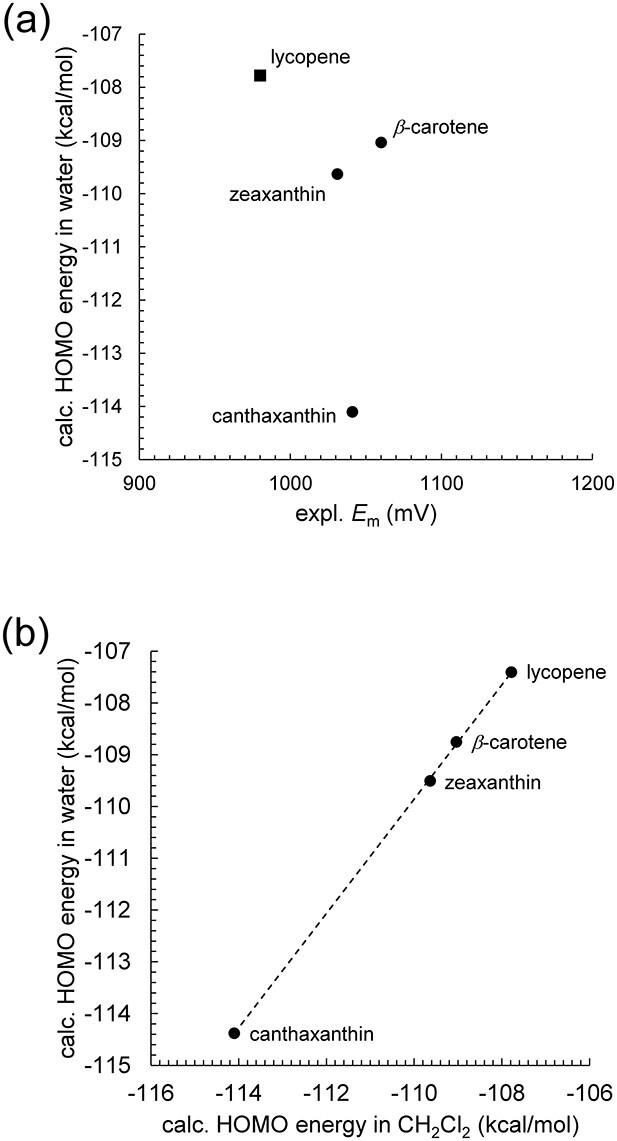
HOMO energy levels of four carotenoids: canthaxanthin, lycopene, *β*-carotene, and zeaxanthin. (a) Correlation with *E*_m_ values reported in micelles. Closed circles represent experimentally measured *E*_m_ values obtained in triton X-100 micelles (canthaxanthin, *β*-carotene, and zeaxanthin), and the closed square represents the *E*_m_ value obtained in mixed triton X-100/triton X-45 micelles (lycopene). (b) Correlation with HOMO energy levels calculated in CH_2_Cl_2_. The dotted line represents the linear correlation (coefficient of determination *R*^2^ = 1.00).

*E*_m_ values of carotenoids have also been reported from micelle-based measurements using tryptophan and its cation radical as a redox mediator in pulse radiolysis (Edge et al. 2000, Burke et al. 2001). These authors argued that electrochemical measurements in organic solvents were difficult to interpret and not biologically relevant, and emphasized that their micelle-based values represented *E*_m_ values in “a biologically relevant environment” (Edge et al. 2000). However, the *E*_m_ values they obtained for carotenoids, including canthaxanthin, lycopene, *β*-carotene, and zeaxanthin, were all clustered within a narrow range of 1020 ± 40 mV *versus* NHE (Edge et al. 2000, Burke et al. 2001). Moreover, the relative *E*_m_ ordering among these carotenoids differs strikingly from those measured in CH_2_Cl_2_ (Liu et al. 2000, Niedzwiedzki et al. 2005) (Fig. 6a). In particular, as demonstrated in the present study (Table 1) and in studies by Niedzwiedzki et al. (2005), the *E*_m_ value of zeaxanthin should be higher than that of *β*-carotene due to the hydroxyl substituents in zeaxanthin, which stabilize the HOMO through electron-withdrawing effects and make oxidation less favorable. In contrast, in micelles, their reported *E*_m_ value of zeaxanthin (1031 mV) is ~30 mV lower than that of *β*-carotene (1060 mV) (Burke et al. 2001). Although these micellar *E*_m_ values should be regarded as relevant measurements in the Triton environments, such discrepancies suggest that micellar conditions introduce environmental effects that modify the apparent *E*_m_ values, making them differ from the intrinsic ordering expected from HOMO energies.

Notably, for the four carotenoids whose *E*_m_ values have been reported in both micelles and CH_2_Cl_2_ (i.e. canthaxanthin, lycopene, *β*-carotene, and zeaxanthin), the calculated HOMO energy levels in water are highly correlated with those in CH_2_Cl_2_ (coefficient of determination *R*^2^ = 1.00, Fig. 6b). This strong correlation suggests that the relative *E*_m_ ordering observed in CH_2_Cl_2_ should also hold true in water, and accordingly in micelles. Indeed, quinones provide a precedent: although their absolute *E*_m_ values differ between DMF *versus* SCE (Prince et al. 1983) and water *versus* NHE (Swallow 1982), the relative *E*_m_ ordering remains highly correlated between the two solvents (*R*^2^ = 0.97), and the *E*_m_ differences among individual quinones are also well preserved (i.e. not clustered as in the micellar measurements of carotenoids) (Kishi et al. 2017). This demonstrates that solvent-dependent *E*_m_ shifts affect absolute scales but not intrinsic trends, suggesting that carotenoids should likewise preserve their relative *E*_m_ ordering between CH_2_Cl_2_ and water. The fact that the reported micellar *E*_m_ values deviate from this ordering implies that micelle-based measurements likely reflect additional environmental factors intrinsic to Triton micelles, rather than only the intrinsic redox properties of the isolated carotenoids. A related concern was raised by He and Kispert, who reported that no oxidation or reduction waves could be detected for *β*-carotene in Triton X-100 micelles using cyclic voltammetry, likely due to the presence of a hydrophobic barrier that inhibits direct electron transfer at the electrode interface (He and Kispert 1999).

Nevertheless, one intriguing feature of the micelle-based data is that, although the HOMO of canthaxanthin is clearly the most stabilized among the four carotenoids investigated, its reported *E*_m_ value is nearly identical to those of *β*-carotene and zeaxanthin. In contrast, lycopene, the only carotenoid among the four with a longer polyene chain, exhibits the lowest *E*_m_ value (Figs. 1 and 6a). This observation suggests that, in micellar environments, *E*_m_ values are relatively insensitive to energetic differences associated with the terminal *β*-ionone rings at both ends of the polyene chain, but are instead more sensitive to the length of the conjugated polyene backbone. A plausible explanation is that the terminal regions of carotenoids are more solvent-exposed in micelles, where the high dielectric constant of water screens the influence of their local polarity on *E*_m_. As a result, the length of the conjugated polyene chain becomes the dominant determinant of *E*_m_, leading to the convergence of the measured values (within 1030−1060 mV) for *β*-carotene, zeaxanthin, and canthaxanthin, which share the same conjugation length, relative to lycopene (980 mV). This interpretation provides a physical rationale for why micelle-based measurements tend to yield nearly identical *E*_m_ values for carotenoids with the same conjugation length. It also explains why the reported *E*_m_ ordering in micelles deviates from both the *E*_m_ ordering in CH_2_Cl_2_ and from the ordering expected from HOMO energy levels. These results suggest that micelle-derived *E*_m_ values are best interpreted as “values in nonionic surfactant environments”, rather than as “reference values in water”.

## Conclusions

*E*_m_ values measured in CH_2_Cl_2_ (Liu et al. 2000) are highly correlated with calculated HOMO energy levels (Fig. 2), enabling the determination of *E*_m_ values for carotenoids whose experimentally measured values have not been reported or where discrepancies exist among measurements, such as spheroidene, 15-*cis*-spheroidene, 15-*cis*-*β*-carotene, antheraxanthin, and violaxanthin (Table 1).

For carotenoids associated with type-II reaction centers, all-*trans* isomers are slightly stronger electron donors than their 15-*cis* counterparts, indicating that the functional significance of *cis* isomerization lies mainly in pigment orientation and optimized protein-pigment contacts rather than in intrinsic *E*_m_ values. For the xanthophyll-cycle carotenoids, the present study demonstrates a stepwise increase in *E*_m_ from zeaxanthin to antheraxanthin to violaxanthin, consistent with their chemical substituents.

The reported micellar *E*_m_ values (Burke et al. 2001) show clear inconsistencies with both calculated HOMO energy levels in water and *E*_m_ values measured in CH_2_Cl_2_ (Fig. 6), indicating that the micellar *E*_m_ values fail to capture the intrinsic redox chemistry of carotenoids. Instead, it seems likely that in micellar environments, *E*_m_ values are relatively insensitive to energetic differences associated with the terminal *β*-ionone rings at both ends of the polyene chain, but are instead more sensitive to the length of the conjugated polyene backbone. Thus, micelle-derived *E*_m_ values can be interpreted as “values in nonionic surfactant environments,” rather than “reference values in water.”

Overall, these results provide a unified framework for the carotenoids involved in type-II reaction centers and associated antenna complexes, clarify long-standing uncertainties in their *E*_m_ values, and offer a molecular-level explanation of how carotenoid chemistry regulates the balance between photoprotection and light harvesting in photosynthesis.

## Methods

As demonstrated previously for quinones (Kishi et al. 2017), the one-electron redox reaction of a molecule A between its oxidized (A_ox_) and reduced (A_red_) states in aqueous solution, the redox potential *E*_m_ relative to the NHE is defined as:


(2) \begin{equation*} {E}_{\mathrm{m}}=\hbox{--} \frac{\varDelta Gaq}{nF} \end{equation*}

where ΔG_aq_ is the free energy difference between A_ox_ and A_red_ in solution (i.e. *ΔG*_aq_ = *G*_aq_(A_red_) – *G*_aq_(A_ox_) – *G*_NHE_), *n* is the number of electrons involved in the reaction (i.e. *n* = 1 in the present case), and *F* is the Faraday constant. Note that in the present case, A_ox_ and A_red_ are the oxidized (Car^•+^) and charge-neutral (Car) states, respectively. *ΔG*_aq_ can also be approximated as:


(3) \begin{equation*} {\varDelta G}_{\mathrm{aq}}={k}^{{\prime}}{\varDelta E}_{\mathrm{QM}/\mathrm{PCM}}+{C}^{{\prime}} \end{equation*}

where *k’* is the scaling factor, *ΔE*_QM/PCM_ is the energy difference between A_ox_ and A_red_ in the solvent phase [i.e. *ΔE*_QM/PCM_ = *E*_QM/PCM_(A_red_) – *E*_QM/PCM_(A_ox_)], calculated using a quantum chemical approach with the polarizable continuum model (PCM) method, and *C′* is a constant (Matsui et al. 2012, Hasegawa et al. 2017, Kishi et al. 2017). Substituting Equation (3) into Equation (2) gives:


(4) \begin{equation*} {E}_{\mathrm{m}}={k}^{"}{\varDelta E}_{\mathrm{QM}/\mathrm{PCM}}+{C}^{"} \end{equation*}

where *k”* is an effective scaling factor and *C″* is a constant (Matsui et al. 2012, Hasegawa et al. 2017, Kishi et al. 2017). These constants can be empirically determined when a sufficient number of experimentally measured *E*_m_ values are available for similar molecular groups.

Although this approach requires evaluation of both reduced and oxidized states, subsequent studies have shown that equivalent accuracy can be maintained by calculating only one redox state: the LUMO for one-electron reduction or the HOMO for one-electron oxidation. In the present work, we employed this updated approach, previously applied to quinones (Ishikita and Saito 2020). In this scheme, the HOMO energy level, *E*_HOMO_, is associated with *E*_m_ for one-electron oxidation (e.g. Watanabe and Kobayashi 1991, Mandal et al. 2020):


(5) \begin{equation*} {E}_{\mathrm{m}}={kE}_{\mathrm{HOMO}}+C \end{equation*}

where *k* is a scaling factor and *C* is a constant. To determine *k* and *C*, we calculated *E*_HOMO(CH2Cl2)_ [and *E*_HOMO(water)_] for carotenoid molecules whose experimentally measured *E*_m_(Car/Car^•+^) values are reported in dichloromethane (CH_2_Cl_2_) (Liu et al. 2000) [and water (Edge et al. 2000; Burke et al. 2001)]. All calculations were performed using the Gaussian program (Frisch et al. 2004). Geometry optimizations were carried out with restricted density-functional-theory (B3LYP/6–31++G**). Solvent effects were modeled with the PCM method, using default dielectric constants (SCRF=Dichloromethane for *ε* = 8.93 and SCRF=Water for *ε* = 78.3553). Atomic partial charges for *β*-carotene and spheroidene were calculated based on the optimized geometries using the CHELPG (Breneman and Wiberg 1990) method. For *β*-carotene, the charges were symmetrized with respect to the C15 = C15’ bond.

## Supplementary Material

pcp-2025-e-00279-File009

## Data Availability

The data underlying this article are available in the article and in its online supplementary material.
