# Supplementary material for: Redox potentials of carotenoids associated with type-II photosynthetic reaction centers
Source: Plant Cell Physiol. 2025 Dec 12;67(3):367–76. doi: 10.1093/pcp/pcaf167 (PMC13078154; doi:10.1093/pcp/pcaf167)
Supplement: pcp-2025-e-00279-File009 [file pcp-2025-e-00279-file009.pdf]

Table S1. Calculated E_{HOMO} (kcal/mol).

carotenoid	E_{HOMO} in CH_2Cl_2	E_{HOMO} in water
α-carotene	-109.4275	
canthaxanthin	-114.3792	
8'-apo-β-caroten-8'-al	-115.2351	
8'-apo-β-caroten-8'-oic acid	-114.5718	
echinenone	-111.3577	
fucoxanthin	-116.1268	
isozeaxanthin	-110.2753	
lycopene	-107.4076	
rhodoxanthin	-113.4599	
[type-II reaction centers]		
spheroidene	-108.7410	
15-*cis*-spheroidene	-109.6007	
β-carotene	-108.7517	-142.5512
15-*cis*-β-carotene	-109.6697	
cryptoxanthin	-109.1382	
[xanthophyll cycle]		
zeaxanthin	-109.5066	
antheraxanthin	-110.1090	
violaxanthin	-111.5209	
[tyrosine]		
Tyr-OH		-103.8150
Tyr-O^-		-109.0360

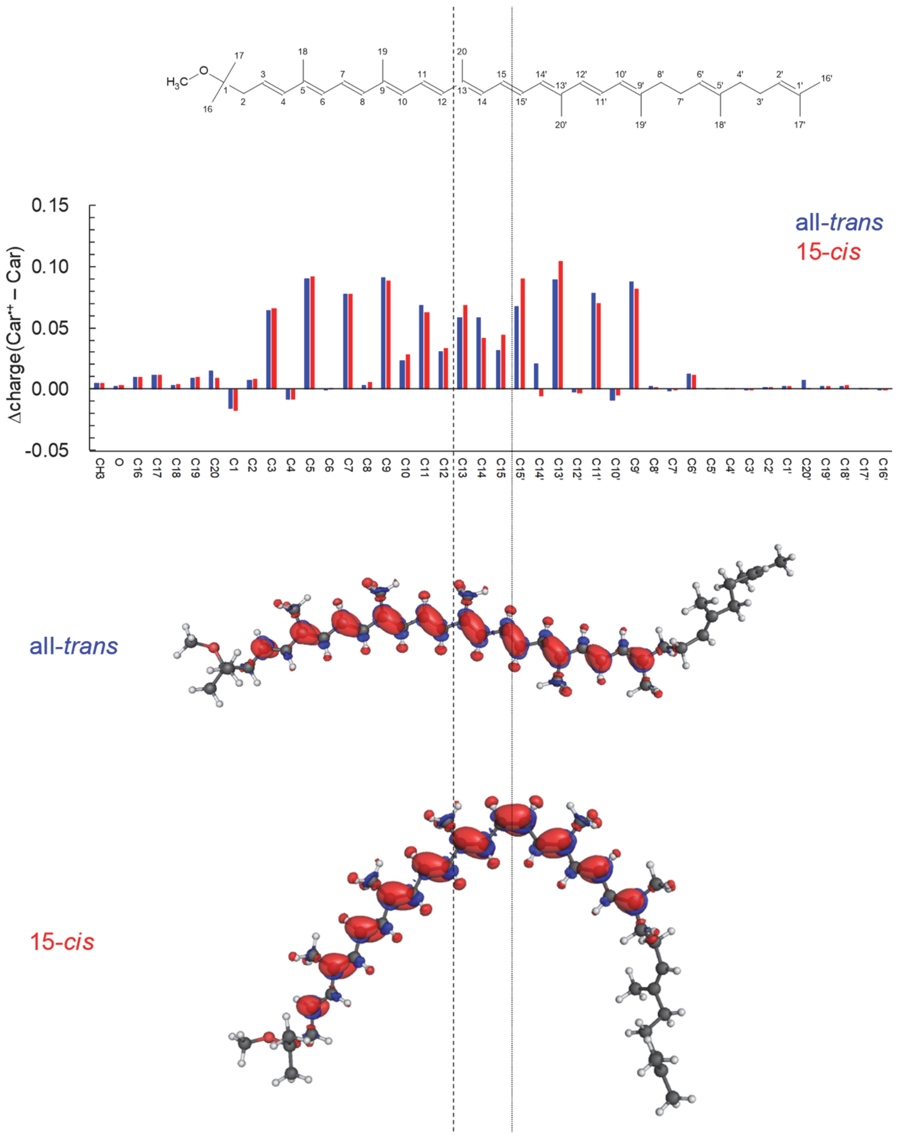

Figure S1. Redistribution of atomic charge and electronic density upon one-electron oxidation in spheroidene. (Top) Molecular structures of spheroidene with atom numbering. (Middle) Changes in atomic partial charges upon one-electron oxidation (Δcharge($Car^{\bullet+}$ – Car)) for the all-*trans* (blue) and 15-*cis* (red) isomers. (Bottom) Electronic density difference ($Car^{\bullet+}$ – Car) for the all-*trans* and 15-*cis* isomers. Red and blue surfaces represent regions of increased and decreased electron density, respectively. The vertical dotted line indicates the center of conjugation. The vertical solid line indicates with the *cis* C15=C15' moiety.
